# Effects of Vitamin D Status and Supplements on Anthropometric and Biochemical Indices in a Clinical Setting: A Retrospective Study

**DOI:** 10.3390/nu11123032

**Published:** 2019-12-12

**Authors:** Myriam Abboud, Xiaoying Liu, Flavia Fayet-Moore, Kaye E. Brock, Dimitrios Papandreou, Tara C. Brennan-Speranza, Rebecca S. Mason

**Affiliations:** 1 Department of Physiology & Bosch Institute, School of Medical Sciences, Faculty of Medicine and Health, University of Sydney, Sydney NSW 2006, Australia; Myriam.abboud@zu.ac.ae (M.A.); xliu0534@uni.sydney.edu.au (X.L.); kaye.brock@sydney.edu.au (K.E.B.); tara.speranza@sydney.edu.au (T.C.B.-S.); 2 Department of Health Sciences, Zayed University, P.O. Box 144534 Dubai, UAE; Dimitrios.Papandreou@zu.ac.ae; 3 School of Molecular Bioscience, University of Sydney NSW 2006, Australia & Nutrition Research Australia, Sydney, NSW 2000, Australia; flavia@nraus.com

**Keywords:** vitamin D deficiency, 25-hydroxyvitamin D, vitamin D supplements, weight loss, low-density lipoprotein (LDL) cholesterol, high-density lipoprotein (HDL) cholesterol, triglycerides (TG), blood pressure

## Abstract

Context: Obesity and low vitamin D status are linked. It is not clear that weight loss through lifestyle intervention is influenced by vitamin D status. Objective: The aim of this study was to investigate the effect of baseline vitamin D status and vitamin D supplementation on weight loss and associated parameters for participants on a weight loss program in a primary care setting. Design: A retrospective analysis of clinical records of patients who underwent an individually tailored weight loss program at a single dietetic clinic in Sydney, Australia. Setting: Primary care centers. Patients: 205 overweight and obese men and women aged from 18 to 50 years. Interventions: Patients were referred to a dietetic clinic for a weight loss program. Patients with low serum 25-hydroxyvitamin D (25(OH)D) concentrations at baseline were advised to increase sun exposure and take multivitamins supplemented with 2000 IU or 4000 IU per day of vitamin D3, according to the preference of their primary care physician. Main outcome measures: Clinical parameters of weight, height, waist circumference, and serum 25(OH)D, as well as blood pressure and fasting lipid profile were collected from both baseline and three-month follow-up consultations. Results: Subjects with sufficient baseline 25(OH)D levels (≥50 nmol/L) experienced significantly greater weight loss (−7.7 ± 5.9 kg vs. −4.2 ± 3.3 kg) and reductions in BMI (−2.6 ± 1.8 kg/m^2^ vs. −1.5 ± 1.1 kg/m^2^) and waist circumference (−5.2 ± 3.5 cm vs. −3.1 ± 3.1 cm) as compared with those who were vitamin D insufficient at baseline (*p* < 0.001 for all). Vitamin D insufficient patients who were supplemented with daily 2000 IU or 4000 IU vitamin D experienced significantly greater decreases in weight (−5.3 ± 3.6 kg vs. −2.3 ± 1.6 kg), BMI (−1.9 ± 1.2 kg/m^2^ vs. −0.8 ± 0.6 kg/m^2^) and waist circumference (−4.2 ± 3.4 cm vs. −1.2 ± 1.3 cm) as compared with those not supplemented (*p* < 0.001 for all). We also observed a greater decrease in low-density lipoprotein (LDL) cholesterol (−0.4 ± 0.5 mmol/L vs. −0.2 ± 0.5 mmol/L) in subjects insufficient at baseline and supplemented as compared with those insufficient at baseline and not supplemented (*p* < 0.01). Conclusion: In a weight loss setting in a dietetic clinic, adequate vitamin D status at baseline, or achieved at three months through supplementation, was associated with significantly greater improvement of anthropometric measures. The study has implications for the management of vitamin D status in obese or overweight patients undergoing weight loss programs.

## 1. Introduction

Vitamin D has multiple pleiotropic functions beyond its traditional role in calcium homeostasis, as well as bone and muscle function [1]. Actions of the vitamin D hormone, calcitriol, have been demonstrated in many tissues, including adipocytes [2] and the cardiovascular system [3]. The first evidence of a relationship between vitamin D and body fat was described in 1972 by Mawer et al. [4]. Inadequate vitamin D status, obesity, and chronic noncommunicable disease often cluster [1,3,5,6,7]. They are important public health issues that contribute significantly to modern healthcare costs, morbidity, and mortality [8,9]. Many studies support the proposal that obesity could be driving low serum 25(OH)D concentrations mainly due to decreased bioavailability of vitamin D through sequestration in body fat compartments [4,10,11,12,13]. There is limited human research which indicates that vitamin D could potentiate weight loss and improvements in metabolic markers [14,15]. A recent randomized controlled trial in postmenopausal women reported that while supplementation with vitamin D did not alter weight loss or associated parameters overall as compared with a placebo group, women in the supplemented group who reached 25(OH)D concentrations of ≥32 ng/mL (≥80 nmol/L), had greater improvement in several measured weight loss parameters as compared with those women whose final 25(OH)D concentrations were below 80 nmol/L [16].

In this study, we hypothesized that overweight and obese patients presenting adequate 25(OH)D levels will have a greater reduction in body weight, body mass index (BMI), and waist circumference as compared with those with inadequate vitamin D levels while undergoing a three-month clinic-specific individually tailored weight loss management program. Furthermore, we expect that vitamin D repletion of those who were insufficient at baseline, through short-term daily vitamin D supplementation would enhance weight loss, decrease waist circumference, and improve biochemical markers. This was investigated using clinic records of a population of overweight and obese men and premenopausal women who participated in an individually tailored three month weight loss program.

## 2. Study Design and Population

This study is a retrospective analysis of a clinical databank that was recorded in a health giver-receiver setting. The Human Ethics Committee at the University of Sydney approved the research protocol (Protocol 2013/206). Between September 2011 and March 2013, a total of 935 patients who attended three medical centers in Sydney, Australia, were referred to a dietetic clinic (established by author MA) to assist with a program for weight loss, under the Chronic Disease Management Plan of Medicare Australia [17]. This care plan entitled each patient to five consultations with a dietician or another allied health professional. Under an agreed protocol, patients had blood taken for 25(OH)D and blood lipid measurements at the initial visit with the primary care physician. These patients were seen by the dietician fortnightly for the first month and then monthly after that. Thus, the initial visit was the first visit, then, after two weeks (second visit), then, another two weeks (third visit), then, after one month (fourth visit), then, after one month (fifth visit). This fifth visit coincided with the three-month follow-up, when the follow-up blood for testing was taken. The referring doctors differed in their approach to management of patients who had 25(OH)D concentrations less than 50 nmol/L at baseline. Some referring doctors advised their patients to take supplements of vitamin D3 at 2000 IU per day, and some at 4000 IU per day. The remaining patients with low baseline 25OHD were advised to increase their sun exposure and take multivitamins (with only small amounts, 40 IU of vitamin D). The dietician performed anthropometric measures at each visit. Weight was measured using the same scales, which were calibrated monthly. Height and waist circumference were measured at the initial and final visits. Waist circumference was measured with the patient standing, at a level midway between the iliac tubercle and lower lateral rib margin, and hip circumference was measured at the level of the iliac tubercle. 

For the individually tailored weight loss protocol, at the initial consultation with the dietician, each patient’s daily estimated energy requirement (EER) was calculated using the Harris-Benedict equation [18] and physical activity factors (see more detailed information in the supplementary Tables S1–S3). Overweight and obese individual caloric goals were calculated to be 300 and 500 Kcal/day, respectively, less than their EER. Once the EER was calculated, a meal plan was designed by the dietitian and given to the participant at the initial consultation, and adherence was checked via a 24-h recall method during each of the follow-up visits. The reported intake was relatively compliant with the prescribed energy intake. The participants were not seen by any exercise physiologist and did not undertake any major changes in physical activity that could have altered their energy needs.

As part of continuing care, a report was sent to the referring primary care physician requesting a follow-up on 25(OH)D and other biochemical markers at three months from the initial consultation, which coincided with the final dietary consultation. This blood test was performed on the same day as the final dietary consultation.

All 935 records of patients who were referred to this program between September 2011 and March 2013 were examined. Of these, 676 records were excluded based on the following predetermined exclusion criteria: a history of diabetes mellitus, polycystic ovary syndrome, parathyroid disorder, kidney or liver disease, osteopenia or osteoporosis, or current pregnancy, or taking any medication known to affect body weight (such as steroids) or supplements such as calcium or vitamin D (>400 IU of vitamin D2 or vitamin D3, not prescribed as part of this intervention). A further 47 patients were excluded as they did not complete the follow-up blood test at three months. There were seven subjects in the group which had sufficient 25(OH)D concentrations (≥50 nmol/L) at baseline, who received vitamin D supplements. These were also excluded from the analysis.

Records of 205 healthy men and premenopausal women between the ages of 18 and 50 were coded for analysis. Clinical parameters including blood pressure; fasting lipid profile, i.e., total cholesterol (TC), low-density lipoprotein (LDL) cholesterol, high-density lipoprotein (HDL) cholesterol, and triglycerides (TG), and serum 25 hydroxyvitamin D (25(OH)D; as well as anthropometric measurements (weight, height, and waist circumference) were collected from both baseline and three-month follow-up consultations. Patients reported sun exposure frequency at baseline and exercise levels.

Overweight and obesity were classified according to BMI (overweight 25 to 29.9 kg/m^2^ and obesity ≥30 kg/m^2^) and waist circumference (overweight men 94.0 to 101.9 cm, women 80.0 to 87.9 cm, obesity men ≥102.0 cm, and women ≥88.0 cm).

### Biochemistry

Plasma levels of cholesterols and triglycerides were determined by standard laboratory methods and were all performed by Laverty Laboratory, North Ryde, Sydney, Australia. They were measured using an enzyme-based Siemens platform, where LDL was calculated in accordance with the Friedewald equation (18). Normal ranges for lipid profile were provided by the commercial laboratory: TC (3.5–5.4 mmol/L), LDL (2.1–4 mmo/L), HDL (>=1 mmol/L), TG (0.1–2 mmol/L). Plasma 25(OH)D concentrations were all determined at the Laverty Laboratory using the Diasorin Siemens chemiluminescent assay and vitamin D insufficiency was defined as 25(OH)D level <50 nmol/L [19]. The assay characteristics are described in [20].

## 3. Statistical Analysis

All analyses were performed using SPSS for Windows (version 17.0 SPSS, Inc., Chicago, IL, USA). Analyzed data were collected from a clinical setting with intention-to-treat approach. Differences in anthropometric and blood parameters between patients who had sufficient baseline 25(OH)D, and insufficient baseline 25(OH)D with or without prescription of vitamin D supplementation, were assessed by one-way ANOVA followed by Tukey’s post-test. Comparisons for the within-group changes in Table 1 were made using paired Student t-tests. Correlations were assessed by calculating Pearson correlation coefficients. LOESS plots [21] were calculated by the SPSS program. Multivariate analysis of changes in weight, BMI, and waist circumference were regressed against 25(OH)D concentrations at follow-up using stepwise linear regression models using the following independent variables: 25(OH)D values at follow-up, adjusting for age, sex, season of baseline appointment, sun exposure, and exercise and were split by prescription of vitamin D supplements. In the initial analyses, the subjects who were vitamin D insufficient at baseline (25(OH)D <50 nmol/L) and supplemented with 2000 IU vitamin D3 per day, were analyzed separately from those who were supplemented with 4000 IU/day. There were no differences between these groups in terms of baseline parameters, except for baseline 25(OH)D which was significantly lower at 31 ± 13 nmol/L in the subjects who were prescribed 4000 IU per day, as compared with 39 ± 16 nmol/L in those prescribed 2000 IU per day (*p* < 0.02). For ease of data presentation and statistical power, these supplemented groups have been combined.

## 4. Results

### 4.1. Subject Characteristics

As shown in Table 1, there was a significant overall reduction in weight, BMI, waist circumference, systolic blood pressure, LDL, and triglycerides after the three-month weight loss program.

There were 70 men and 135 women whose records were included in the study. Although the men were significantly older (mean 39 years vs. 37 years, *p* = 0.007) and had significantly higher weight, waist circumference, blood pressure, LDL, and triglyceride values, and lower HDL, at baseline, they were not significantly different from the women in terms of baseline BMI, 25(OH)D, or the proportion who were vitamin D sufficient (see Supplementary Table S1). At three months, there were again no significant differences between males and females in terms of BMI, 25(OH)D concentrations, or the proportion who were vitamin D sufficient, but differences in the other parameters persisted (Supplementary Table S2). Sex had no significant effect on changes in weight, BMI, waist circumference, 25(OH)D concentration, or total cholesterol over the three-month period of analysis (*p* values of >0.05 for all). For this reason, in Table 1 and subsequent tables, data for male and female subjects have been combined.

Consistent with the high prevalence of vitamin D insufficiency with obesity, the mean baseline serum 25(OH)D concentration was insufficient (45 ± 19 nmol/L) and the mean baseline BMI classified subjects as obese overall. At baseline, 3% of the baseline subjects were in the normal weight range, 50% were overweight, and 47% were obese. After three months on a weight loss program and supplementation with vitamin D for some individuals, the mean serum 25(OH)D level was significantly higher than that of the baseline at 54 ± 17 nmol/L (*p* < 0.001) with a three month median and interquartile range of 55 and 23 nmol/L, respectively, while the mean BMI and waist circumference were significantly lower than that of the baseline (Table 1). After three months on the program, 22% of subjects were normal weight, 45% were overweight, and 33% were obese.

### 4.2. Effect of Vitamin D Status and Supplementation on Anthropomorphic Measures and Other Parameters

Baseline serum 25(OH)D was significantly higher in the sufficient group as compared with the deficient group (64 ± 11 vs. 33 ± 10 nmol/L, *p* < 0.001, Table 2). After three months on the program, 25(OH)D concentrations were similar to baseline values in both vitamin D sufficient and vitamin D insufficient individuals not given supplemental vitamin D, despite advice to increase sun exposure and take multivitamins (Table 2).

At three months, the subjects who were vitamin D sufficient at baseline experienced significantly greater body weight loss, BMI decrease, and waist circumference reduction than those with baseline vitamin D deficiency (*p* < 0.001 for all, Table 3). The season of baseline appointment did not affect the weight changes at three months.

Of the subjects with baseline vitamin D insufficiency, 60% received vitamin D supplements, which raised 25(OH)D concentrations significantly (*p* < 0.001, Table 2 and Table 3). The initially low vitamin D status subjects who received supplemental vitamin D also experienced significantly greater body weight loss, as well as a greater reduction in BMI and waist circumference than those who were not supplemented (*p* < 0.001 for all parameters, Table 3). Vitamin D supplementation resulted in a significant decrease in LDL-cholesterol levels in this group as compared with the non-supplemented subjects (*p* < 0.01, Table 3). However, there were no significant differences in the changes in total cholesterol, HDL-cholesterol, TG, diastolic, or systolic BP between the subjects who received supplements and those who did not. Even with supplementation, the reductions in weight, BMI, and waist circumference in the initially vitamin D deficient group were significantly lower than those of the initially vitamin D sufficient group (*p* < 0.001).

The increase in 25(OH)D concentrations in those subjects who were initially vitamin D deficient and prescribed 2000 IU/day (*n* = 40) was 15 ± 7 nmol/L, significantly lower than the 37 ± 15 nmol/L increase in 25(OH)D concentrations in those subjects prescribed 4000 IU/day of vitamin D (*n* = 30) (*p* < 0.02 as compared with the lower dose group). Those subjects who were vitamin D deficient at baseline and who were prescribed 4000 IU of vitamin D3 daily, lost significantly more weight (−6.9 vs. −4.4 kg; *p* < 0.001), significantly reduced their BMI to a greater extent (−2.5 vs. −1.5 kg/m^2^; *p* = 0.001), and reduced their waist circumference more effectively (−5.3 vs. −3.3 cm; *p* < 0.001) than those who were told to take 2000 IU daily. The dose of supplemental vitamin D did not significantly affect changes in LDL, HDL, and total cholesterol.

**Figure 1:**
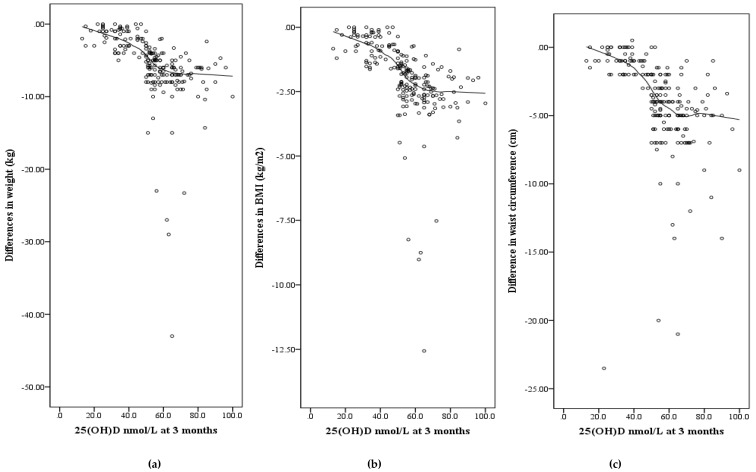
Shows changes in weight (**a**), BMI (**b**), and waist circumference (**c**) between baseline and 3 months plotted against serum 25(OH)D concentrations at 3 months for all 205 participants.

The relationship between changes in weight, BMI, and waist circumference, and concentrations of 25(OH)D at three months, for all patients are shown in Figure 1a–c. In general, the largest decreases in each of these measurements were in patients whose follow-up 25(OH)D was above 50 nmol/L. After adjusting for age, sex, season, sun exposure, and exercise, a stepwise multivariate regression model for changes in weight, BMI, and waist circumference showed that body weight loss, and decreases in BMI and waist circumference reduction were all significantly associated with 25(OH)D concentrations as the independent variable at three months (Table 4).

## 5. Discussion

The results presented here, derived from clinical dietetic practice, showed that higher baseline vitamin D status was associated with significantly greater weight loss and larger reductions in BMI and in waist circumference during a weight loss program. The analysis also showed that, on this individually tailored weight loss program, vitamin D supplementation for those participants who were vitamin D insufficient at baseline, enhanced weight loss, BMI reductions, and reductions in waist circumference.

Although there have been a number of studies on the effects of vitamin D status and vitamin D supplementation on weight loss and related parameters over time (reviewed in [22,23,24]), most of these studies did not include subjecting participants to an individually tailored weight loss regimen. Overall, the results of meta-analyses of studies which did not include a specific weight loss regimen, showed that while better vitamin D status before or during a randomized controlled trial of vitamin D supplementation predicted greater weight or fat loss over time in a few studies, overall there was no effect of vitamin D supplementation on body mass index or fat mass, even in larger studies of longer duration, such as that of Sneve et al. [25]. A recent publication reported a significant reduction in waist circumference in subjects in a randomized controlled trial of 16 weeks of high dose vitamin D supplements vs. placebo [26]. The subjects were of Asian ethnicity living in Melbourne, Australia and this was a secondary analysis of the trial, which did not include instructions on weight loss. Vitamin D status has been reported to affect weight loss and related parameters in some trials involving weight loss regimens [27,28], but not in the majority of such studies, although body weight or composition were secondary outcomes in most of these studies [22,23]. A recent review by Bassatne et al. (2019) found no clear evidence for a beneficial effect of vitamin D supplementation on cardiometabolic parameters in obese individuals and the authors concluded that Vitamin D supplementation had no effect on weight loss [24], although data on biochemical parameters and weight loss are very scarce. In a study of children with variants in the fat mass and obesity-associated gene (FTO), which affect adiposity in an age-dependent manner, it was reported that FTO genotype re9939609 was associated with a significant weight gain in children who were vitamin D insufficient, defined as <75 nmol/L, but no significant genetic effects were observed in vitamin D sufficient children [29].

There have been very few randomized controlled trials which were designed to examine the effect of vitamin D supplementation on weight loss and body composition during a weight loss program. Zittermann et al. [14] randomized 200 healthy overweight men and women, with mean 25(OH)D of 30 nmol/L, who were on an individually tailored weight loss program, to either vitamin D3 supplementation (83 ug = 3332 IU/day) or a placebo. At the end of 12 months, 25(OH)D concentrations were 55 nmol/L higher in the supplemented group, but there was no significant difference in weight loss over this time between the two groups. Mason et al. [16] enrolled 218 overweight or obese women over 50 years, with baseline 25(OH)D between 25 and 80 nmol/L (mean 53 nmol/L) and randomized them to a weight loss program with 2000 IU/d of vitamin D3 or to weight loss with a placebo. Although changes in weight, BMI, and waist circumference were similar between the groups overall at 12 months, those women whose 25(OH)D rose to above 80 nmol/L (≥32 ng/mL) lost significantly more weight and reduced their waist circumference and percent body fat to a significantly greater extent than women who achieved a 25(OH)D of <80 nmol/L [16].

In our study there was no effect of vitamin D status at baseline or of vitamin D supplementation on changes in systolic or diastolic blood pressure, or changes in total cholesterol, HDL, or triglycerides. The decrease in LDL, however, was significantly greater in the baseline sufficient subjects as compared with the deficient group as a whole, and in the latter group, was significantly greater in those supplemented with vitamin D (Table 3). Mason et al. [16] did not report lipid data but did show a significant decrease in C-reactive protein in the vitamin D supplemented group amongst those with complete pill counts (97%) as compared with a placebo. Although Zitterman et al. [14] showed no effect of vitamin D supplementation on weight loss in their 12-month randomized controlled trial, vitamin D supplementation resulted in significant decreases in triglyceride and TNF-α concentrations, but a significant increase in LDL cholesterol. There have been inconsistent results reported in several studies, overall showing no effect on cardiovascular or inflammatory markers with vitamin D supplementation, although these were not carried out in conjunction with an individually tailored weight loss program [30].

The current analysis examined data was neither randomized nor blinded, and therefore the potential effects of conscious or unconscious bias cannot be discounted. Another limitation is the use of 24 h recall to assess adherence to the dietary plan, which is known to be biased by misreporting [31,32], specifically underreporting. Due to this limitation, which is difficult to overcome, it is not possible to properly assess the role that different energy intakes affected weight loss. The initial data included all records of those who attended the clinic for the standardized chronic disease management plan in the period September 2011 to March 2013. The criteria for exclusion were decided in advance of the data collection and, as expected, excluded a very large number of subjects. Nevertheless, the cohort of patients whose data were analyzed were all part of a standardized chronic disease management plan established through a clinical partnership between three medical centers and a dietetics clinic. The main variable was the referring primary care physician’s preference for improvement of vitamin D status in the patients (either advice to increase sun exposure + multivitamins (in Australia, multivitamins have negligible vitamin D), or supplementation with either 2000 IU/d or 4000 IU/d of vitamin D3). Based on the data in Table 2, the groups were well matched. As patients were not randomized, there is a possibility that the effects of vitamin D supplementation were chance findings. The patient numbers in each of these groups were limited, so it is difficult to make definitive interpretations, although the apparently greater weight loss in the group supplemented with 4000 IU/day, who achieved higher 25(OH)D concentrations than those supplemented with 2000 IU/day, is intriguing and may at least provide preliminary data for future studies. Critical for vitamin D studies that are interpretable [33], the mean baseline 25(OH)D concentrations in the deficient group in this study were both well below 50 nmol/L, nearly 60% of subjects were deficient at baseline, and, although tablet counts were not performed, those who were supplemented increased their 25(OH)D concentrations by an average of 25 nmol/L (baseline deficient group).

A strength of this analysis is that the female and male subjects in the clinical cohort were relatively young, with a mean age <40 y. BMI and 25(OH)D concentrations did not differ between men and women at baseline or at three months (Supplementary Tables S1 and S2). Sex had no significant effect on changes in BMI, waist circumference, or 25(OH)D, and therefore males and females were combined for further analysis to increase statistical power. Nevertheless, there were effects of sex on other parameters tested. A further limitation of the analysis is that the data in Table 2 and Table 3 were not adjusted for sex or other factors, unlike Table 4, which may have influenced the outcomes reported.

As indicated by the multivariate analysis (Table 4), the effect of the achieved 25(OH)D concentration on changes in weight, BMI, and waist circumference was present even after adjustment for age, sex, season, and physical activity, indicating that these factors did not explain the effect of vitamin D supplementation. The mechanisms which might explain an effect of vitamin D status on weight loss are poorly understood. It is generally agreed that high body fat is likely to cause low vitamin D status, due to sequestration of vitamin D in adipose tissue [11]. A Mendelian randomization approach has also reported that a higher BMI leads to low vitamin D status and not the other way around [13]. Possible explanations about how vitamin D status could influence adiposity are still speculative. Since low vitamin D results in increased parathyroid hormone, this increased parathyroid hormone could increase calcium influx into adipocytes, which in turn, could inhibit lipolysis and stimulate lipogenesis [34]. Adipocytes express 1α-hydroxylase [35] and actively accumulate 25(OH)D [36]. As reviewed by Ding et al. [2] the vitamin D receptor (VDR) is expressed in adipocytes and preadipocytes and its expression has been shown to be dynamically upregulated during adipogenesis. Paradoxically, VDR knockdown in adipocytes inhibited adipogenesis and VDR knockout mice showed less body fat and were resistant to diet-induced obesity [37]. Effects of vitamin D status on central control of appetite are also plausible, considering the wide expression of the VDR in the central nervous system, including the hypothalamus [38].

Decreases in anthropomorphic parameters in our study were generally greater in those subjects who were initially vitamin D sufficient, regardless of later supplementation. In those who were vitamin D deficient at baseline, supplementation with vitamin D improved the weight loss parameters measured. After initiation of a supplementation regimen, it takes approximately three months for 25(OH)D to reach a plateau [39,40], and therefore the initially deficient patients would have remained relatively deficient for part of the study period.

On the basis of these findings, we cannot discard the hypothesis that sufficient serum 25(OH)D concentrations could be a determinant of the success of a weight loss program. Since vitamin D is synthesized endogenously, it would not be possible to show an effect of supplementation in subjects who are already “sufficient” by whatever criteria this is determined. Indeed, in this study, a large proportion (60%) of the clinic population were vitamin D deficient at baseline. The results of the study support the proposal that correction of low levels of 25(OH)D could benefit weight loss and the lipid profile of some overweight or obese patients, leading to the conclusion that, in clinical practice, it is important to individualize vitamin D therapy and educate the patient on the need for supplementation only when necessary.

## Figures and Tables

**Table 1:** Baseline and follow-up characteristics for all subjects.

	Baseline N = 205	Follow Up N = 205	
Parameters	Mean ± SD	Mean ± SD	*p* value
Weight, kg	88.5 ± 18.3	82.9 ± 17.6	<0.001
BMI, kg/m^2^	31 ± 5.3	29.1 ± 5.2	<0.001
Waist circumference, cm	97.7 ± 14.1	93.7 ± 13.8	<0.001
25(OH)D, mmol/L	45.2 ± 18.7	54.0 ± 16.8	<0.001
BP systolic, mm Hg	126.1 ± 14.3	122.9 ± 11.4	<0.001
BP diastolic, mm Hg	77.8 ± 10.4	78.0 ± 8.5	NS
Total cholesterol, mmol/L	5.7 ± 3.7	5.1 ± 0.9	0.02
LDL, mmol/L	3.2 ± 0.9	2.9 ± 0.8	<0.001
HDL, mmol/L	1.5 ± 0.4	1.4 ± 0.3	NS
Triglycerides, mmol/L	1.6 ± 1.1	1.4 ± 0.8	<0.001

25(OH)D indicates serum 25-hydroxyvitamin D; HDL, high-density lipoprotein; LDL, low-density lipoprotein; and BP, blood pressure. Data are shown as mean values ± SD data from 205 subjects, except for total cholesterol and triglycerides (204), LDL (194), and HDL (197). *p* values show differences between baseline and follow-up values, NS, non-significant *p* > 0.05.

**Table 2:** Baseline values for anthropomorphic measures, lipids, and blood pressure, and baseline and follow-up values for 25(OH)D with or without the three month supplementation with vitamin D. Values are presented as means ± SDs.

	Sufficient at Baseline	Insufficient at Baseline			ANOVA *p*-Value ^#^
Parameters	Suf	Total	Non-Supplemented (Insuf-NonSup)	Supplemented (Insuf-Sup)	
**N**	82	123	48	75	
Age (years)	37 ± 8.2	38 ± 7.5	37 ± 7.4	38 ± 7.6	0.485
% Female	60%	70%	67%	73%	
Weight, kg	89 ± 17	88 ± 19	88 ± 17	87 ± 21	0.764
BMI, kg/m^2^	31 ± 5	31 ± 5	31 ± 4	31 ± 6	0.534
Waist, cm	97 ± 14	98 ± 14	98 ± 13	98 ± 15	0.922
Baseline 25(OH)D nmol/L	64 ± 11 ^a,b^	33 ± 10	34 ± 10	32 ± 10	**<0.001**
Follow-up 25(OH)Dnmol/L	62 ± 11 ^a,b^	49 ± 18	35 ± 11	57 ± 16 ^c^	**<0.001**
BP-systolic, mmHg	126 ± 14	126 ± 15	127 ± 13	125 ± 16	0.793
BP-diastolic mmHg	77 ± 10	78 ± 11	78 ± 10	78 ± 11	0.908
Total cholesterol mmol/L	5.9 ± 5.8	5.5 ± 1.1	5.5 ± 1.2	5.5 ± 1.0	0.704
LDL mmol/L	3.2 ± 0.8	3.2 ± 1.0	3.1 ± 1.1	3.3 ± 0.9	0.647
HDL mmol/L	1.5 ± 0.4	1.5 ± 0.4	1.5 ± 0.5	1.5 ± 0.4	0.950
triglyceride mmol/L	1.4 ± 0.9	1.7 ± 1.2	1.8 ± 1.4	1.6 ± 1.1	0.203

^#^ For differences between sufficient at baseline, Insuf-NonSup and Insuf-Sup. The total column is presented for information. ^a^ *p* < 0.05 Suf vs. Insuf-NonSup (Tukey’s post hoc test); ^b^ *p* < 0.05 Suf vs. Insuf-Sup (Tukey’s post hoc test); ^c^ *p* < 0.05 Insuf-NonSup vs. Insuf-Sup (Tukey’s post hoc test). Significant *p* values are shown in bold.

**Table 3:** Changes in 25(OH)D, weight, BMI, waist circumference, lipids, and blood pressure with or without the three month supplementation with vitamin D. Values are presented as means ± SDs.

	Sufficient at Baseline	Insufficient at Baseline			ANOVA *p*-Value ^#^
Change (Δ) in Parameters	Suf	Total	Non-Supplemented (Insuf-NonSup)	Supplemented (Insuf-Sup)	
N	82	123	48	75	
Δ25(OH)D, mmol/L	−1.7 ± 7 ^b^	16 ± 17	1.0 ± 4.8	25 ± 16 ^c^	**<0.001**
Δweight, kg	−7.7 ± 5.9 ^a,b^	−4.2 ± 3.3	−2.3 ± 1.6	−5.3 ± 3.6 ^c^	**<0.001**
ΔBMI, kg/m^2^	−2.6 ± 1.8 ^a,b^	−1.5 ± 1.1	−0.8 ± 0.6	−1.9 ± 1.2 ^c^	**<0.001**
Δwaist circum-ference, cm	−5.2 ± 3.5 ^a^	−3.1 ± 3.1	−1.3 ± 1.3	−4.2 ± 3.4 ^c^	**<0.001**
Δsystolic BP, mmHg	−4.8 ± 9.2	−2.2 ± 11	−3.1 ± 8.4	−1.6 ± 12	0.165
Δdiastolic BP, mmHg	−0.2 ± 8.6	0.4 ± 9.1	1.4 ± 8.2	−0.2 ± 9.6	0.553
ΔTotal Cholesterol mmol/L	−1.0 ± 5.6	−0.3 ± 0.5	−0.2 ± 0.4	−0.4 ± 0.5	0.378
ΔLDL mmol/L	−0.4 ± 0.4 ^a^	−0.26 ± 0.5	−0.1 ± 0.4	−0.4 ± 0.5 ^c^	**0.002**
ΔHDL mmol/L	−0.1 ± 0.2	−0.1 ± 0.2	−0.1 ± 0.2	−0.1 ± 0.2	0.380
Δtriglycerides mmol/L	−0.2 ± 0.4	−0.2 ± 0.7	−0.2 ± 0.9	−0.2 ± 0.5	0.920

^#^ For differences between sufficient at baseline, Insuf-NonSup and Insuf-Sup. The total column is presented for information. ^a^ *p* < 0.05 Suf vs. Insuf-NonSup (Tukey’s post hoc test); ^b^ *p* < 0.05 Suf vs. Insuf-Sup (Tukey’s post hoc test); ^c^ *p* < 0.05 Insuf-NonSup vs. Insuf-Sup (Tukey’s post hoc test). Significant *p* values are shown in bold.

**Table 4:** Multivariate regression model ^#^ for the association between changes in weight, BMI, and waist circumference with concentrations of 25(OH)D at 3 months.

	Insufficient at Baseline *N* = 123		All Subjects *N* = 205	
Change (Δ) in Parameters over 3 Months	Non-Supplemented (Insuf-NonSup)	Supplemented (Insuf-Sup)	Non-Supplemented	Supplemented
N	48	75	130	75
Δweight, kg	−0.45 ***	−0.61 ***	−0.47 ***	−0.60 ***
ΔBMI, kg/m^2^	−0.45 ***	−0.63 ***	−0.46 ***	−0.63 ***
Δwaist circumference, cm	−0.75 ***	−0.29 ***	−0.55 ***	−0.29 ***

Data expressed as adjusted beta coefficients ^#^ Adjusted for age, sex, season, sun exposure, and exercise. Difference = (follow-up – baseline) values, *** *p* < 0.001.

## Supplementary Materials

The following are available online at https://www.mdpi.com/2072-6643/11/12/3032/s1, Table S1: Baseline characteristics of men and women; Table S2: 3-month follow up characteristics of men and women; Table S3. The Harris–Benedict equations.
