# Supplementary material for: Effects of Vitamin D Status and Supplements on Anthropometric and Biochemical Indices in a Clinical Setting: A Retrospective Study
Source: Nutrients. 2019 Dec 12;11(12):3032. doi: 10.3390/nu11123032 (PMC6950220; doi:10.3390/nu11123032)
Supplement: Supplementary file 1 [file nutrients-11-03032-s001.pdf]

Supplementary Table S1. Baseline characteristics of men and women

	Men		Women		
Parameters	n	Mean ±SD	n	Mean (95% CI)	**t-test p-value**
Age, y	70	39.4 ±6.6	135	36.6 ± 8.2	0.007 *
Weight, Kg	70	98.7 ±17.6	135	83.2 ±16.4	<.001 *
BMI, Kg/m^2	70	31.3 ±4.7	135	30.9± 5.5	0.55
Waist circumference, cm	70	103.5 ± 11.3	135	94.6±14.5	<.001 *
25(OH)D, mmol/l	70	48 ± 18	135	44 ± 19	0.15
%25(OH)D Sufficient	70	47	135	36	0.14
BP Systolic, mm Hg	70	131.4 ± 13.7	135	123.4 ± 13.9	<.001 *
BP Diastolic, mm Hg	70	80.7 ± 10.2	135	76.3 ± 10.3	0.003 *
Total Cholesterol, mmol/l	70	5.67 ± 1.14	135	5.66 ± 4.55	0.98
LDL, mmol/l	63	3.5 ± 0.9	131	3.1 ± 0.9	0.002 *
HDL, mmol/l	69	1.3 ± 0.33	131	1.6 ± 0.42	<.001 *
Triglycerides, mmol/l	70	2.1±1.5	135	1.3 ± 0.7	<.001 *

Supplementary Table S2. 3-month follow up characteristics of men and women

	Men		Women		
Parameters	n	Mean ±SD	n	Mean ±SD	**t-test p-value**
Weight, Kg	70	91.8 ±16.6	135	78.3±16.3	<.001 *
BMI, Kg/m^2	70	29.1 ±4.5	135	29.0 ±5.5	0.90
Waist circumference, cm	70	99.1 ± 11.2	135	91.0 ±14.2	<.001 *
25(OH)D, mmol/l	70	56 ± 16	135	53 ± 17	0.18
%25(OH)D Sufficient	70	69	135	63	0.45
BP Systolic, mm Hg	70	126.8 ±10.6	135	120.8 ±11.3	<.001 *
BP Diastolic, mm Hg	70	79.8 ± 7.76	135	77.0 ±8.8	0.022 *
Total Cholesterol, mmol/l	70	5.3 ±1.0	134	5.0±0.9	0.05 *
LDL, mmol/l	65	3.12 ± 0.86	132	2.8 ± 0.83	0.017 *
HDL, mmol/l	66	1.2 ± 0.3	132	1.5 ±0.3	<.001 *
Triglycerides, mmol/l	70	1.6 ± 1.0	134	1.2 ± 0.6	0.002 *

25(OH)D indicates Serum 25-hydroxyvitamin D; HDL:high-density lipoprotein; LDL:low-density lipoprotein; BP:blood pressure. Data are shown as mean values (95% CI).

Supplementary Table S3. The Harris–Benedict equations

**Lifestyle**	**Example**	**PAL**	total energy expenditure (TEE)
Sedentary or light activity	Office worker getting little or no exercise	1.53	BMR x 1.53
Active or moderately active	Construction worker or person running one hour daily	1.76	BMR x 1.76
Vigorously active	Agricultural worker (non mechanized) or person swimming two hours daily	2.25	BMR x 2.25

The Harris–Benedict equations

Men	BMR = (10 × weight in kg) + (6.25 × height in cm) - (5 × age in years) + 5
Women	BMR = (10 × weight in kg) + (6.25 × height in cm) - (5 × age in years) - 161

Ref: Mifflin MD, St Jeor ST, Hill LA, Scott BJ, Daugherty SA, Koh YO (1990). "A new predictive equation for resting energy expenditure in healthy individuals". The American Journal of Clinical Nutrition. 51 (2): 241–7. doi:10.1093/ajcn/51.2.241. PMID 2305711.
